# Heterologous Protein Secretion in Lactobacilli with Modified pSIP Vectors

**DOI:** 10.1371/journal.pone.0091125

**Published:** 2014-03-10

**Authors:** Ingrid Lea Karlskås, Kristina Maudal, Lars Axelsson, Ida Rud, Vincent G. H. Eijsink, Geir Mathiesen

**Affiliations:** 1 Department of Chemistry, Biotechnology and Food Science, Norwegian University of Life Sciences, Ås, Norway; 2 Nofima – Norwegian Institute for Food, Fisheries and Aquaculture Research, Ås, Norway; Charité, Campus Benjamin Franklin, Germany

## Abstract

We describe new variants of the modular pSIP-vectors for inducible gene expression and protein secretion in lactobacilli. The basic functionality of the pSIP system was tested in *Lactobacillus* strains representing 14 species using pSIP411, which harbors the broad-host-range *Lactococcus lactis* SH71_rep_ replicon and a β-glucuronidase encoding reporter gene. In 10 species, the inducible gene expression system was functional. Based on these results, three pSIP vectors with different signal peptides were modified by replacing their narrow-host-range *L. plantarum* 256_rep_ replicon with SH71_rep_ and transformed into strains of five different species of *Lactobacillus*. All recombinant strains secreted the target protein NucA, albeit with varying production levels and secretion efficiencies. The Lp_3050 derived signal peptide generally resulted in the highest levels of secreted NucA. These modified pSIP vectors are useful tools for engineering a wide variety of *Lactobacillus* species.

## Introduction

Lactic acid bacteria (LAB) are widely used in the food industry, and are also increasingly applied as probiotics and as producers of enzymes and metabolites, mainly due to their GRAS (Generally Regarded As Safe) status. LAB can contribute to the quality, preservation and safety of fermented food products [1], one reason being that LAB inhibit spoilage microbes by production of lactic acid and bacteriocins [2], [3]. Several LAB are natural inhabitants of the GI-tract of animals and humans, and have potential as *in situ* delivery vectors of antigens and other medically interesting proteins [4]. Some species of lactobacilli have been studied extensively, and several *Lactobacillus* strains are known to exert probiotic effects on human health [5]. Recently, several studies have demonstrated that some lactobacilli have immune-stimulatory properties, which may be relevant when applying these bacteria for in situ delivery of molecules to mucosal surfaces [6], [7]. Given the importance of the lactobacilli in (functional) food, their potential as cell factory and delivery vehicle, and the apparent functional variation between genus members, it is imperative that versatile tools for protein expression and secretion are available for a variety *Lactobacillus* species.

In the past two decades several expression systems for production of heterologous proteins in LAB have been developed [8], [9], including systems that lead to secretion of the overexpressed protein [10]–[12]. Secretion of heterologous proteins is challenging and often leads to the use of heterologous DNA from distantly related microbes, coding for signal peptides (SPs). We have previously developed the pSIP expression vectors [12], [13] which allow inducible protein expression using the regulatory machinery naturally involved in bacteriocin production in *Lactobacillus sakei* [3]. The original vectors have been developed further to allow secretion of the expressed heterologous proteins [14], [15]. Genome-wide screening of SPs from *L. plantarum*, using NucA as model protein, revealed large variation between SPs, both in terms of expression yield (i.e. the amount of protein produced) and secretion efficiency (i.e. the amount of produced protein that is actually secreted) [15].

The pSIP system has been successfully applied for intracellular expression [16], [17], secretion [18], [19] and surface anchoring [20], [21] of a variety of proteins in *L. plantarum* and *L. sakei.* Generally, the use of the pSIP vectors has been limited to derivatives containing the narrow host range 256_rep_ replicon [22], although a broad-host-range derivative, pSIP411, was constructed early in the development of the pSIP system [13]. Moreover, the applicability of the previously cloned *L. plantarum* SPs in other *Lactobacillus* species has not yet been explored. Notably, *Lactobacillus* spp. display considerable variation in their probiotic, cell-wall, and molecular properties [23], which is relevant for their application in different environments or products. Furthermore, host factors can have effects on heterologous protein expression [24]. All in all, this highlights the importance of testing and adopting the pSIP expression vectors to a wider host range, and of verifying the usefulness of previously selected SPs in other *Lactobacillus* species.

The goal of the present study was to analyze the applicability of the pSIP expression system in lactobacilli, focusing on secretion of heterologous proteins in other *Lactobacillus* species than *L. plantarum.* Basic functionality of the pSIP system (i.e. inducible gene expression) was first tested in several lactobacilli using the broad-host range vector pSIP411. The original 256_rep_ replicon in selected pSIP secretion vectors was then replaced with the replicon present in pSIP411 to enable expression and secretion of NucA in five different species of *Lactobacillus*: *L. rhamnosus*, *L. brevis*, *L. gasseri* and *L. curvatus* and *L. plantarum*. These species represent different phylogenetic groups within the genus *Lactobacillus* [25] and include both human and food isolates (Table 1). *L. plantarum, L. gasseri* and *L. rhamnosus* are known for their immunomodulatory properties [26]–[28] and *L. rhamnosus* GG, also known as LGG, is marketed as probiotic. *L. brevis* and *L. curvatus* are often found in fermented foods, and may have probiotic properties (e.g. [29]). *L. plantarum* and *L. gasseri* have been used extensively for *in situ* delivery of mucosal vaccines [30], [31]. For secretion, we evaluated three SPs derived from the *L. plantarum* proteins Lp_3050, Lp_0373 and Lp_2578, which had previously shown different abilities to direct secretion of NucA in *L. plantarum* [15].

**Table 1:** Bacterial strains used in this study.

Strains	Comments, isolation	References or source
*Lactococcus lactis* IL1403	Subcloning host strain	[52]
*Lactobacillus (L.) plantarum* WCFS1	Human saliva, secretion host	[44]
*L. brevis* ATCC 8287	Green olives, secretion host	ATCC
*L. rhamnosus* GG	Human GI tract, secretion host	Valio Ltd, Finland [46]
*L. curvatus* DSM 20019^ T^	Milk, secretion host	DSMZ
*L. gasseri* ATCC 33323^T^	Human GI tract, secretion host	[45]
*L. acidophilus* ATCC 4356^T^	Human GI tract	ATCC
*L. coryniformis* NCIMB 9711^T^	Silage	NCIMB
*L. farciminis* MF1292	Dry fermented sausage	[53]
*L. helveticus* ATCC 15009^T^	Emmental cheese	ATCC
*L. johnsonii* MF2395	Human GI tract	Nofima, Norway
*L. paracasei* NCIMB 700151^T^	Milk	NCIMB
*L. pentosus* DSM 20314^T^	Corn silage	DSMZ
*L. pentosus* MF1300	Dry fermented sausage	[53]
*L. plantarum* NC8	Silage	[12]
*L. plantarum* MF1298	Dry fermented sausage	[53]
*L. reuteri* DSM 20016^T^	Human GI tract	DSMZ
*L. reuteri* ATCC PTA 6475	Human mother’s milk	BioGaia, Sweden
*L. sakei* DSM 20017^T^	Saké	DSMZ
*L. sakei* Lb790	Meat	[12]
*L. sakei* 23K	Dry fermented sausage	INRA, France [54]

## Materials and Methods

### Bacterial Strains and Growth Conditions

The bacterial strains and plasmids used in this study are listed in Tables 1 and 2. *E. coli* TOP10 (Invitrogen, Carlsbad, CA, USA) cells were grown in BHI broth (Oxoid Ltd., Hampshire, England) at 37°C with shaking. *Lactococcus lactis* IL1403 cells were grown in M17 broth (Oxoid) supplemented with 0.5% glucose (GM17 medium) at 30°C without agitation. Lactobacilli were grown in MRS broth (Oxoid) at different temperatures (25, 30 and 37°C) without agitation for the initial pSIP functionality test (see below). Thereafter, *L. plantarum*, *L. gasseri*, and *L. rhamnosus* cells were grown in MRS broth at 37°C without agitation, and *L. curvatus* and *L. brevis* were grown in MRS broth at 30°C without agitation. Solid media were prepared by addition of 1.5% (w/v) agar to the broth. When required, antibiotics were added as follows: for *E. coli*, erythromycin (200 µg/mL); for *L. lactis*, erythromycin (10 µg/mL); for all *Lactobacillus* species, erythromycin (5 µg/mL).

**Table 2:** Plasmids used in this study.

Plasmids	Comments	References or source
pSIP411	Initial screening and source of SH71_rep_	[13]
pEV	pLp_2578sAmyA derivative, no sp, no AmyA (negative control)	[20]
pLp0373NucA	NucA fused to the sp_Lp_0373_, with 256_rep_, Em^R^	[15]
pLp3050NucA	NucA fused to the sp_Lp_3050_, with 256_rep_, Em^R^	[15]
pLp2578NucA	NucA fused to the sp_Lp_2578_, with 256_rep_, Em^R^	[15]
pLp0373NucA-SH71	NucA fused to the sp_Lp_0373_, with SH71_rep_, Em^R^	This work
pLp3050NucA-SH71	NucA fused to the sp_Lp_3050_, with SH71_rep_, Em^R^	This work
pLp2578NucA-SH71	nucA fused to the sp_Lp_2578_, with SH71_rep_, Em^R^	This work

### Plasmid Purification and Preparation of Competent Cells

Plasmids from *E. coli* were purified using the Nucleospin plasmid miniprep kit (Macherey-Nagel GmbH & Co., Düren, Germany). For plasmid isolation from *L. lactis* cells were pretreated with GTE-buffer (50 mM glucose, 25 mM Tris-HCl pH 8.0, 10 mM EDTA, pH 8.0) containing 40 mg/mL lysozyme, 0.8 mg/mL RNase and 80 U/mL mutanolysin prior to the lysis step in the plasmid miniprep protocol. *L. lactis* was used as sub cloning host and was transformed as described by Holo and Nes [32]. *L. reuteri* was transformed according to Ahrné et al. [33]. All other *Lactobacillus* strains were transformed essentially as described by Aukrust et al. [34], but with the following modifications: (1) 4% instead of 1% glycine was used in the preparation of electro-competent *L. coryniformis,* and (2) for *L. acidophilus*, *L. gasseri*, *L. helveticus*, *L. johnsonii*, *L. paracasei* and *L. rhamnosus*, the cells were washed three times in wash buffer (5 mM Na-phosphate, 1 mM MgCl_2_, pH 7.4), and resuspended in E-buffer (0.9 M sucrose, 3 mM MgCl_2_, pH 7.4) before storage.

### Functionality Screen of the pSIP Inducible Gene Expression System

*Lactobacillus* strains (Table 1) were transformed with pSIP411 [13]. Overnight cultures of the transformed strains were inoculated in MRS broth containing 5 µg/mL erythromycin, and incubated at 25, 30 or 37°C (three temperatures tested for each transformant). The cultures were induced by adding the inducing peptide pheromone (SppIP) to 100 ng/mL at OD_600_ 0.3, and allowed to grow over night (approximately 15–20 h, depending on strain and temperature). β-glucuronidase (GUS) activity was determined as described by Axelsson et al. [35].

### Cloning Strategy

To construct the modified secretion vectors, the SH71_rep_ fragment (2 kb) was generated by digesting the pSIP411 vector [13] with *Acc*651 and *Bam*HI and ligated to the larger fragment generated by *Acc*65I/*Bam*HI digestion of plasmids pLp0373NucA, pLp3050NucA and pLp2578NucA to replace the 256_rep_ replicon. This yielded vectors pLp0373NucA-SH71, pLp3050NucA-SH71and pLp2578NucA-SH71. Constructs verified by DNA sequencing were electrotransformed into competent *Lactobacillus* spp.

### SDS-PAGE Analysis and Western Blot Analysis

Overnight cultures of *Lactobacillus* spp. harboring the newly constructed pSIP secretion vectors (Tables 1 and 2) were diluted in MRS medium containing 5 µg/mL erythromycin. The cultures were induced by adding the inducing peptide pheromone at OD_600_ 0.3 as described previously [36]. Cells were harvested 4 hours after induction by centrifugation at 6 000×g for 7 min at 4°C, after which the supernatants were filtered (0.22 µM) and PMSF was added to 1 mM final concentration. The supernatant samples were run on 10% NuPAGE Novex Bis-tris gels using MOPS as running buffer (both Invitrogen). The proteins in the supernatant fractions were visualized using the Pierce Silver Stain Kit for Mass Spectrometry from Thermo Scientific (Rockford, IL) following the manufacturer’s protocol. The cells were washed three times with ice-cold 0.9% (w/v) NaCl. To extract intracellular proteins, washed cells were resuspended in 0.1 M Tris-HCl (pH 8) containing 0.01 M EDTA and 1 M NaCl (TEN buffer; 5% of the harvesting volume), before adding PMSF (1 mM final concentration). The cells were disrupted with glass-beads using a FastPrep-24 instrument (MP Biomedicals, Solon, OH) (speed 6.5, 45 seconds at 4°C).

When western analysis was appropriate, proteins from both the intracellular and extracellular fraction were separated by SDS-PAGE as described above and transferred to a nitrocellulose membrane using the iBlot Dry Blotting System (Invitrogen) according to manufacturer’s recommendations. Rabbit polyclonal anti-NucA antiserum against the peptide EFDKGQRTDKYGRG [15], [37] was obtained from ProSci Inc. (Poway, CA) and used as recommended by the manufacturer. Protein bands were visualized by using a horseradish peroxidase-conjugated (HRP) goat anti-rabbit antibody (Bio-Rad) and the enhanced chemiluminescent kit from Pierce (Roche, IL).

### Plasmid Copy Number Analysis by Quantitative Real-time PCR

All primers used in this study (Table S1) were purchased from Operon Biotechnologies GmbH (Cologne, Germany). Total DNA was isolated and purified from cells harvested 3–4 hours after inductions (see above) using the phenol-chloroform extraction method as previously described [38]. DNA was isolated from two independent cultures of each transformant, and analyzed as independent replicates throughout the real-time PCR procedure. All real time qPCR amplifications were performed using a StepOnePlus™ Realtime PCR- system (Applied Biosystems, Carlsbad, CA).

qPCR reactions were prepared in triplicate for both chromosomal (*groEL*) and plasmid (*eryR*) amplicons. Each reaction included 10 µl 2× Power SYBR® Green® PCR Master Mix (Applied Biosciences), 10 pmol of each primer and 1 µl of DNA template in a total reaction volume of 20 µl. In negative controls the DNA template was replaced with water.

The qPCR program was as follows: initial denaturation at 95°C for 10 min, followed by 40 cycles of 95°C for 15 s, and 60°C for 1 min. After the last cycle, the temperature was increased from 60°C to 95°C at a rate of 0.3°C/s to establish the melting curve. The threshold cycle values (C_t_) were automatically generated by the StepOne software v2.0 (Applied Biosystems) and exported to Excel for further analysis.

The plasmid copy number (PCN) was calculated using the following equation [39]: PCN = (E_c_)^Ctc^/(E_p_)^Ctp^. Here, E_c_, C_tc_ and E_p_, C_tp_ are the amplification efficiency (E) and the threshold cycle (C_t)_ value of the chromosome (c) and plasmid (p) amplicons, respectively. Amplification efficiencies were determined using validation experiments according to Livak and Schmittgen [40], which showed sufficient equivalence between the amplification efficiencies of the chromosomal and plasmid amplicons.

## Results and Discussion

### Host Range of the pSIP411 Vector and Inducible Gene Expression in *Lactobacillus* Species

The pSIP411 vector [13], containing the lactococcal broad-host-range replicon SH71_rep_ [41] was initially screened for functionality in several *Lactobacillus* strains (Table 1) representing another 12 species in addition to *L. plantarum* and *L. sakei*, for which the pSIP system originally was developed [12]. Replication of the pSIP411 vector as an intact plasmid was confirmed in all strains by plasmid preparation and restriction analysis (data not shown), confirming the broad-host-range of SH71_rep_. The testing of gene expression was simplified by using overnight cultures instead of cells in a defined growth phase for GUS activity measurements. Thus, the test only gave a qualitative indication of whether inducible gene expression of *gusA* functioned or not (Table 3). Induction using 100 ng/mL of sppIP, i.e. approximately 10 times the concentration required in *L. plantarum* and *L. sakei* to achieve full induction [13], resulted in appreciable GUS expression in 10 of the 14 *Lactobacillus* species (including *L. plantarum* and *L. sakei*) at various temperatures (25°C, 30°C and 37°C). Importantly, for the ten successful species, the expression system worked at all temperatures compatible with growth, which may be important in applications and production optimization procedures. Notably, functionality did not correlate with phylogenetic relationships; for example, the system did not work in *L. pentosus* (tested in two strains), a species closely related to *L. plantarum.* Similarly, of the three species belonging to the “*acidophilus* complex”, the system failed to work in *L. johnsonii*, but performed well in *L. acidophilus* and *L. gasseri*. After the initiation of this study, others have shown the pSIP system to be applicable in *L. reuteri* [42], [43] and *L. casei* [16]. Notably, the causes of the non-functioning of the pSIP system in some of the strains tested here need further investigation. The lack of GUS activity could be due to failure of SppIP-mediated transcriptional induction, but also to misfolding and/or degradation of the expressed protein.

**Table 3:** Qualitative functionality of the pSIP inducible gene expression system in *Lactobacillus* strains grown at various temperatures.

	Temperature (°C)		
Species (no. of strains)	25	30	37
*L. acidophilus* (1)	+^a^	+^a^	+^a^
*L. brevis* (1)	+	+	+
*L. coryniformis* (1)	–	–	–
*L. curvatus* (1)	+	+	+
*L. farciminis* (1)	+	+	–b
*L. gasseri* (1)	+	+	+
*L. helveticus* (1)	–	–	–
*L. johnsonii* (1)	–	–	–
*L. paracasei* (1)	+	+	+
*L. pentosus* (2)	–	–	–
*L. plantarum* (3)	+	+	+
*L. reuteri* (2)	–c	+	+
*L. rhamnosus* (1)	+	+	+
*L. sakei* (3)	+	+	+

a +, >100 Miller Units (MU) GUS activity; non-induced cultures were <30 MU in all cases.

b poor growth at this temperature.

c no growth at this temperature.

### Modification of pSIP Secretion Vectors Expands their Host Range in Lactobacilli

**Figure 1:**
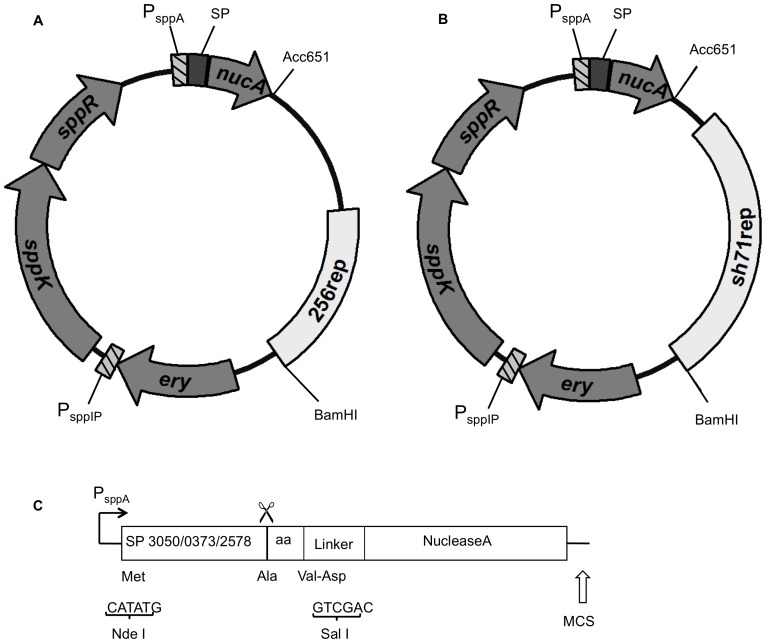
Schematic overview of the vectors used in this study. The 256_rep_ replicon in vector (a) was replaced by the SH71_rep_ replicon, resulting in vector (b). See text for detailed description. The marked genes and fragments encode the following: dark grey, SP (signal peptide); medium grey, Nuclease A (*nucA*) reporter, erythromycin resistance marker (*ery*), histidine kinase (*sppK*), and response regulator (*sppR*); light grey, replicon (256_rep_ or SH71_rep_); striped, inducible promoters P*_sppA_* and P*_sppIP_*. (c) Schematic overview of the secretion cassette used in the pSIP-vectors. The signal peptides (Lp_3050, Lp_0373, or Lp_2578) are followed by the first two amino acids (aa) of their cognate mature protein. These are followed by a two residue linker encoding the amino acids valine and aspartic acid and generating a *Sal*I site, which is fused to the target gene encoding the reporter protein NucA. [14] The scissors indicates the signal peptide cleavage site. MCS indicates a multiple cloning site, which includes the *Acc*651 site used for replicon exchange.

Three pSIP vectors for secretion of *Staphylococcus aureus* nuclease A (NucA) with different *L. plantarum* signal peptides (Lp_3050, Lp_0373 and Lp_2578) were selected from a previously generated genome-wide SP-library [14], [15]. When used in *L. plantarum* these SPs showed varying levels of secreted protein (3050>0373>2578) and secretion efficiencies varying from close to 100% (3050, 0373) to clearly less than 100% (2578) [15]. The host range of these previously developed pSIP vectors were expanded by replacing the 256_rep_ replicon with the SH71_rep_ replicon (Figure 1). The resulting vectors were re-transformed into *L. plantarum* WCFS1 [44], and four other *Lactobacillus* species: *L. gasseri* ATCC 33323T [45], *L. rhamnosus* GG (LGG) [46], *L. brevis* ATCC 8287 and *L. curvatus* DSM 20019. We obtained transformants for all three secretion vectors in all species.

### NucA Secretion in Lactobacilli

**Figure 2:**
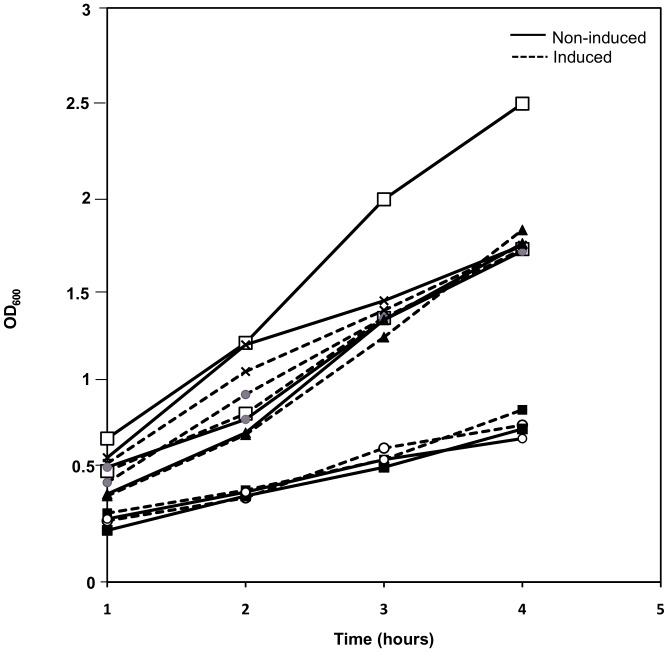
Growth curves for induced (–) and non-induced (–) cells of five different *Lactobacillus* species containing pLp3050NucA-SH71. Optical density (OD_600_) was measured every hour after induction of NucA production. The graphs show *L. plantarum* (▴), *L. gasseri* (□), *L. rhamnosus* (▪), *L. curvatus* (•), and *L. brevis* (○). For comparison, graphs for *L. plantarum* harboring pLp3050NucA (**×**) (256_rep_) are also shown. *L. curvatus* and *L. brevis* species were grown at 30°C, whereas the other species were grown at 37°C.

The effect of NucA production and secretion on growth rate was analyzed by comparing growth of induced and non-induced *Lactobacillus* harboring pLp3050NucA-SH71. Figure 2 shows similar growth rates for induced and non-induced cultures of all species except *L. gasseri*, which shows impaired growth after induction. The reduced growth rate after induction may indicate stress due to heterologous protein production and/or secretion of NucA. Secretion stress is a common problem accompanying heterologous expression in gram-positive bacteria [47], [48]. Figure 2 also shows that *L. brevis* and *L. rhamnosus* generally grew slower than the other lactobacilli.

**Figure 3:**
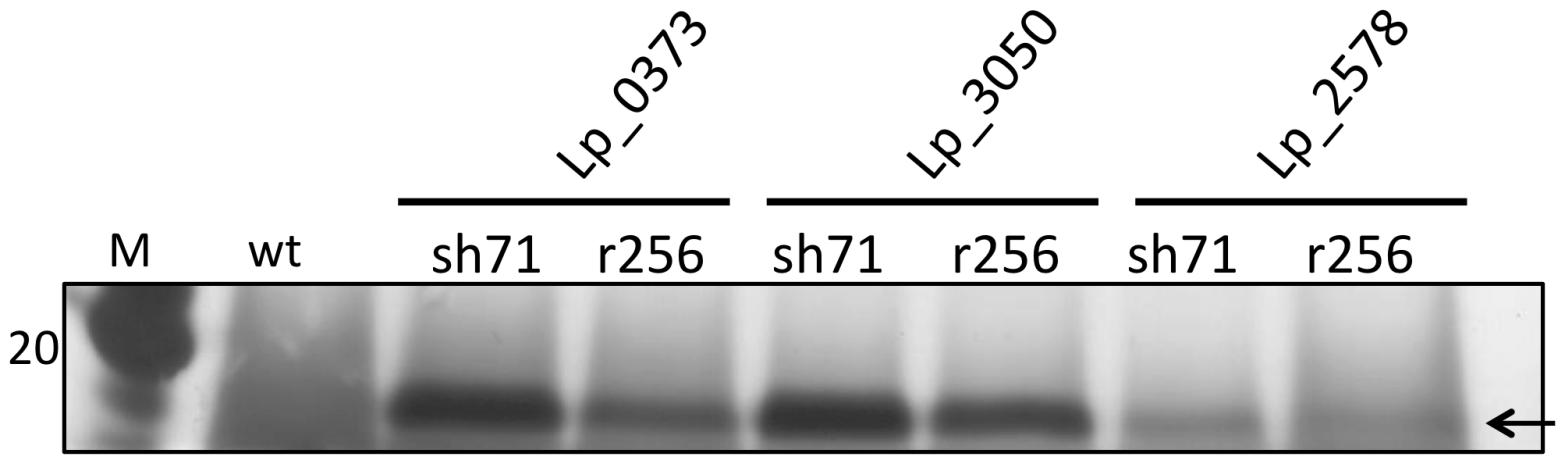
Silver stained SDS-PAGE gel showing NucA in cell free supernatants from *Lactobacillus plantarum* WCFS1 harboring different expression vectors. The vectors differ with respect to the signal peptide (Lp_0373, Lp_3050 or Lp_2578) and the replicon (256_rep_ or SH71_rep_), as indicated in the Figure. The sample size was 15 µl (Lp_3050 and Lp_0373) or 20 µl (Lp_2578). Lane M shows the molecular mass standard (kDa); wt indicates supernatant from *L. plantarum* WCFS1 without expression vector (15 µl).

After exchanging the 256_rep_ replicon with the SH71_rep_ replicon, we first compared the NucA level in the supernatants of induced *L. plantarum* cells harboring the various plasmids. Figure 3 shows that *L. plantarum* harboring vectors containing the SH71_rep_ replicon secreted more NucA compared to transformants harboring the corresponding vectors containing the 256_rep_ replicon. Thus, the change of replicon had a positive effect on the amount of extracellular NucA produced by *L. plantarum*.

**Figure 4:**
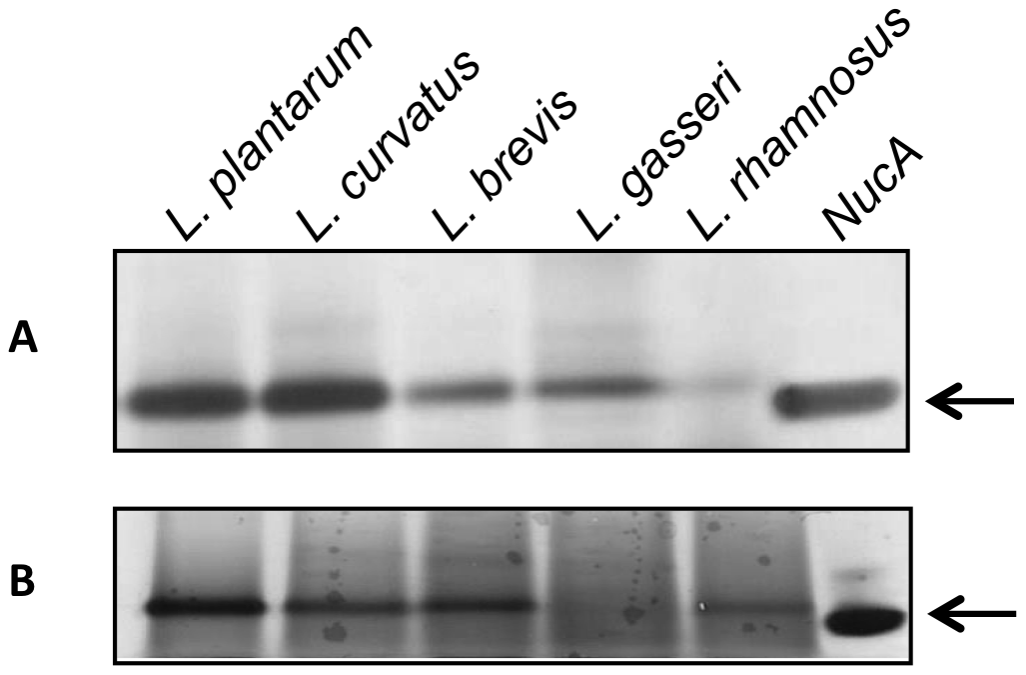
Silver-stained SDS-PAGE gels showing cell-free supernatants of various lactobacilli. The gels show NucA production in induced cultures of five different *Lactobacillus* species harboring (**a**) pLp3050Nuc-SH71 or (**b**) pLp0373Nuc-SH71. The sample size was 15 µl except for *L. rhamnosus* (20 µl). Note that the cultures had different cell densities after the four hour induction period (Fig. 2). The lanes marked NucA contain 0.5 µg NucA standard (Sigma). The arrows indicate NucA.

The ability of *L. plantarum* SPs to drive NucA secretion in other lactobacilli was then examined by SDS-PAGE analysis of cell-free supernatants from induced cultures of the twelve other transformants (four *Lactobacillus* species, three transformants each). Most transformants containing constructs with the Lp_3050 or Lp_0373 SPs produced considerable levels of extracellular NucA (Figure 4A & 4B). The Lp_2578 SP performed poorly, resulting in low extracellular NucA levels in most species (data not shown), similar to or lower than the levels found in *L. plantarum* (Figure 3). All species harboring the vector with the Lp_3050 SP secreted NucA, and this SP generally seemed to give the highest levels of extracellular NucA. *L. rhamnosus* GG was an exception: secretion with the Lp_3050 SP was low and only in this strain the Lp_0373 SP performed better than the Lp_3050 SP. Use of the Lp_0373 SP led to secretion of NucA in all species except in *L. gasseri*, which did not produce any NucA with either the Lp_0373 (Figure 4B) or the Lp_2578 SP (data not shown; confirmed by Western blotting; see below).

**Figure 5:**
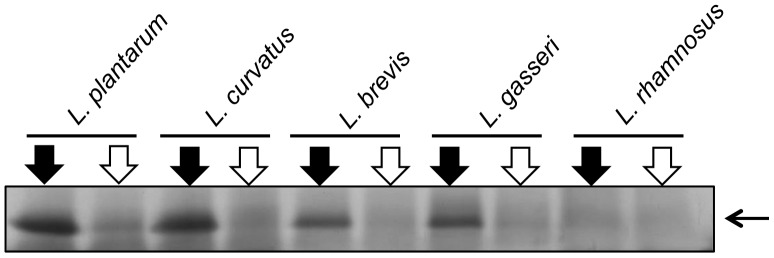
Silver-stained SDS-PAGE gel showing cell-free supernatants of various lactobacilli. The gel shows NucA production in induced (black arrow) and non-induced cultures (white) of five different *Lactobacillus* species harboring pLp3050Nuc-SH71. The sample size was 15 µl species except for *L. rhamnosus* (20 µl). Note that the cultures had different cell densities after the four hour induction period (Fig. 2). The horizontal arrow indicates NucA.

The promoters driving the expression of the gene of interest in the pSIP vectors are known to be strictly regulated in *L. plantarum*, where basal expression from the promoters is low, albeit depending on both the promoter and the gene of interest [12], [49]. On a general note, a well-regulated system may be an advantage for the production of proteins that are detrimental to the host [4]. The level of basal expression was assessed for transformants harboring the Lp_3050 constructs and Figure 5 shows extracellular NucA levels in the supernatants of induced and non-induced cells. The gels show low levels of basal NucA production, in all host species tested. Thus, the regulation of the pSIP system is maintained, although minor differences in the tightness of the regulation may occur that are not detectable on the silver stained SDS-PAGE gels.

### Secretion Efficiency

**Figure 6:**
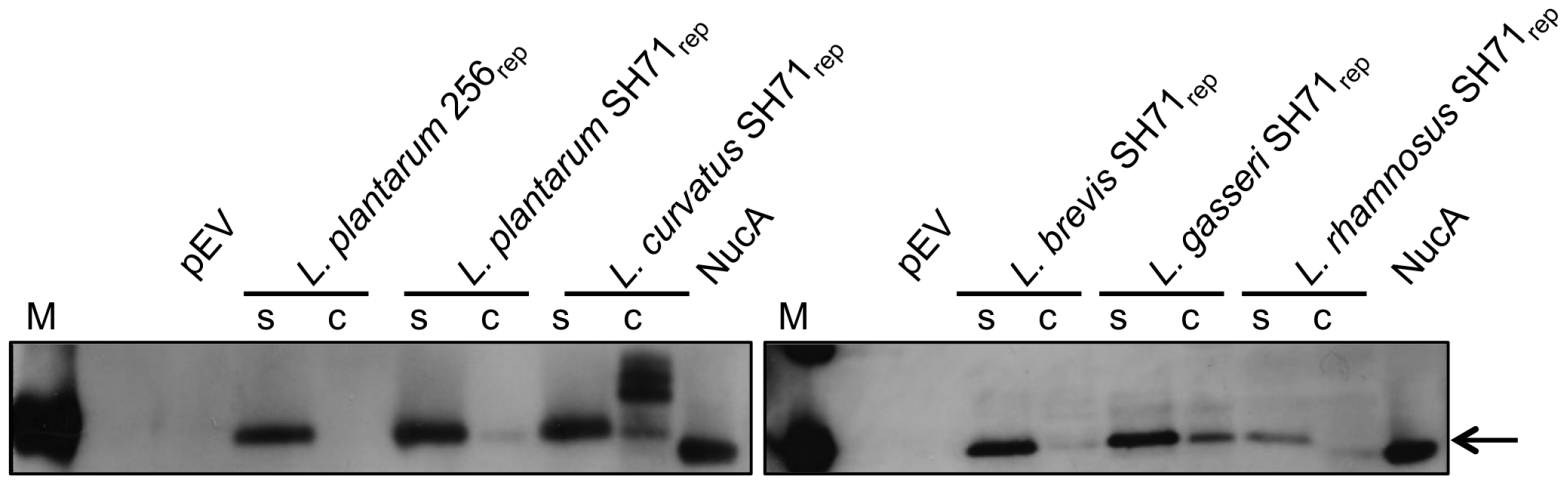
Western blots for analysis of secretion efficiency. The gels show proteins in cell lysates (C) and supernatants (S) of *L. plantarum* containing pLp3050NucA-SH71 or pLp3050NucA (256_rep_ replicon), *L. curvatus*, *L. brevis* and *L. gasseri* containing pLp3050NucA-SH71, and *L. rhamnosus* containing pLp0373NucA-SH71. Lane M, molecular mass standard (20 kDa band); lane NucA contains 0.5 µg NucA standard (Sigma), indicated by the arrow. The lanes marked pEV show supernatants of *L. plantarum* harboring an empty vector without *nucA*. For all the culture-derived samples, the sample size corresponded to 20 µl of the original culture, which was harvested 4 hours after induction.

To analyze the secretion efficiency in the *Lactobacillus* species, levels of NucA in cell lysates and culture supernatants of the best performing transformants were compared using Western blot analysis with a NucA-specific antibody. All fractions showed only one major band, except for the cell lysate of *L. curvatus* (Figure 6; see below). The data indicate secretion efficiencies close to 100% for *L. plantarum, L. brevis and L. rhamnosus*, since cell lysates showed no or very low NucA signals. The cell lysates of *L. gasseri* and especially *L. curvatus* showed considerable NucA levels. The strong bands seen for *L. curvatus* at a mass slightly above the mass of the secreted protein were not observed in cell lysates of non-induced cells (data not shown) indicating that these bands represent non- or incorrectly processed NucA. Taken together, the *L. curvatus* samples show that this strain produced the highest levels of NucA, but that there are limitations in the processing and secretion capacity. Interestingly, these limitations in the secretion pipeline were not accompanied by particular effects of induction on the growth rate of the bacterium (Figure 2). Cell lysates of *L. gasseri* also contained a considerable amount of NucA. In this case however, the retained NucA was correctly processed (Figure 6).

Using Western blots, it was shown that the inability of *L. gasseri* to secrete NucA with the Lp_0373 or Lp_2578 SPs was due to the fact that no NucA was produced at all in these species (data not shown). PCR analysis of the two isolated vectors showed that the erythromycin resistant gene of the pSIP vector was intact, while the NucA gene part of the vector was missing. Thus, in contrast to the pLp3050NucA-sh71 construct, the constructs carrying the other two SPs were not stable in *L. gasseri*. Additional experiments to obtain stable transformants with the other two SPs failed. *L. gasseri* carrying pLp3050NucA-sh71 was the only strain for which induction (i.e. production of NucA) led to a clear reduction in the growth rate (Figure 2). Taken together, these observations (vector instability in two out of three cases; low secretion efficiency and growth inhibition upon induction in the third case), indicate that secretion of NucA leads to major stress in *L. gasseri*.

### Plasmid Copy Number Determination

To further analyze the performance of the newly developed expression vectors plasmid copy numbers (PCN) were determined for the best working vector (in terms of secreted NucA) for each species. Plasmid copy numbers were determined by comparing the genomic *groEL* gene with the plasmid-borne *eryR* gene [50] using real time qPCR. The results (Table 4) show that replacing the 256_rep_ replicon by the SH71_rep_ replicon leads to an almost five-fold increase in copy number in *L. plantarum* (from 2.0 to 9.8). Figures 3 and 6 shows that this increase in copy number is accompanied by an increase in NucA production/secretion, although the band intensities on the gels indicate that this increase is less than five-fold. A comparison between the Western blot analysis (Figure 6) and calculated plasmid copy numbers further shows that the species with the highest (*L. curvatus*) or lowest (*L. rhamnosus*) PCN correspondingly yielded the highest and lowest total amount of NucA, respectively. All in all, the data suggest that an increase in copy number is beneficial for NucA production. Notably, other factors, such as the capacity of the transcription, translation and translocation apparatus also play a role in determining the overall success of a transformant. As an example, in the case of *L. curvatus*, the copy number and total protein level are relatively high, but Figure 6 shows that the secretion apparatus does not seem to be able to keep up with protein production.

**Table 4:** Plasmid copy number (PCN) in lactobacilli harboring vectors with the *NucA* reporter gene.

Strain	Vector	PCN*
*L. plantarum*	pLp3050NucA	2.0±0.2
*L. plantarum*	pLp3050NucA-SH71	9.8±2.8
*L. curvatus*	pLp3050NucA-SH71	14.9±1.3
*L. brevis*	pLp3050NucA-SH71	3.9±1.5
*L. gasseri*	pLp3050NucA-SH71	2.7±0.9
*L. rhamnosus*	pLp0373NucA-SH71	1.3±0.3

*All plasmid copy numbers were calculated from minimum two biological replicates, each analyzed by triplicate qPCR runs. The data shown are the means ± standard deviations.

## Conclusions

The possibility to use the pSIP system for secretion of heterologous proteins in *L. plantarum* has already been explored in several studies [18]–[20]. In the present study, we have modified the pSIP secretion vectors with a broad-host-range replicon, enabling use in different *Lactobacillus* strains. Furthermore, we show that *L. plantarum* SPs function in several of these species, which yields additional tools for genetic engineering of these important food and potentially probiotic bacteria. Importantly, the effectiveness of a SP for secretion of a protein is difficult to predict, and depends on both the protein and the expression strain used. Additionally, effects of SP variation on overall expression levels are common and not easy to rationalize [15], [51]. In line with this, our present data show strain-dependent variation between the SPs, in terms of both total expression levels and secretion efficiency. While one generally should try out several SPs, both the present and previous results indicate that the SPs from Lp_3050 and Lp_0373 are relatively safe bets, definitively in *L. plantarum*, and, as shown above, also in several other lactobacilli.

All in all, the new vectors presented here provide useful tools for modification of a variety of lactobacilli which could be used in the development of these bacteria as delivery vectors for biotechnologically and therapeutically interesting proteins. Potential applications vary from relatively well explored applications of LAB as live vaccine-delivery vectors to fine-tuning LAB probiotic properties. As an example, Park et al. [29] recently showed that combined administration of *L. curvatus* and *L. plantarum* modulates the gut microbiota in mice and leads to reduced obesity. The expression vectors described above allow secretion of heterologous proteins in both these species and could thus be used for in situ delivery of beneficial compounds in the gastro intestinal tract.

## Supporting Information

Table S1 **Oligonucleotide primers used in this study.**
(DOCX)
