# Supplementary material for: Heterologous Protein Secretion in Lactobacilli with Modified pSIP Vectors
Source: PLoS One. 2014 Mar 10;9(3):e91125. doi: 10.1371/journal.pone.0091125 (PMC3948729; doi:10.1371/journal.pone.0091125)
Supplement: Table S1 — Oligonucleotide primers used in this study. (DOCX) [file pone.0091125.s001.docx]

| **Primers** | **Strains** | **Primer Sequence 5’ - 3’** | **Sources or references** | **Accession number** |
| --- | --- | --- | --- | --- |
| Lb_GroEL_F | *L. brevis* | TGAAGAACGTCACTGCTGGGGCTA | This work | AY424330.1 |
| Lb_GroEL_R | *L. brevis* | AGCGGCACCAGTCGCCTTTT | This work | AY424330.1 |
| Lp_GroEL_F | *L. plantarum* | TGGGGTTGCCGTTGTTCGTGTC | This work | NC_004567.2 |
| Lp_GroEL_R | *L. plantarum* | CTTCTTCAACGGCGGCCCGAG | This work | NC_004567.2 |
| Lg_GroEL_F | *L. gasseri* | AGGTGCTGGCTCAAAGGATGCAA | This work | YP_814252.1 |
| Lg_GroEL_R | *L. gasseri* | ACAGCAACACCACCAGCAAGCT | This work | YP_814252.1 |
| Lc_GroEL_F | *L. curvatus* | ACGTCACAGCCGGCGCTAAC | This work | AY424345.1 |
| Lc_GroEL_R | *L. curvatus* | AGCAGCCACTTGGGCAATGGC | This work | AY424345.1 |
| Lr_GroEL_F | *L. rhamnosus* | ACGGTCGAAGTCGCTGGTCG | This work | YP_003171985.1 |
| Lr_GroEL_R | *L. rhamnosus* | AGGCCGTGCAGGCAAGGTTAC | This work | YP_003171985.1 |
| EryR_F |  | CCGTGCGTCTGACATCTAT | [50] |  |
| EryR_R |  | TGCTGAATCGAGACTTGAGTG | [50] |  |
